# Heritage Decorative Wooden Flooring Restoration—Systemotechnical Approach and Risk Analysis

**DOI:** 10.3390/ma19030631

**Published:** 2026-02-06

**Authors:** Michał Juszczyk, Leonas Ustinovichius, Michał Pyzalski, Piotr Buda, Paweł Murzyn

**Affiliations:** 1 Faculty of Civil Engineering, Cracow University of Technology, Warszawska 24, 31-155 Kraków, Poland; 2 Faculty of Civil Engineering, Vilnius Gediminas Technical University, Saulėtekio 11, LT-10223 Vilnius, Lithuania; leonas.ustinovicius@vilniustech.lt (L.U.); michal.pyzalski@agh.edu.pl (M.P.); 3 Faculty of Materials Science and Ceramics, AGH University of Krakow, Al. Mickiewicza 30, 30-059 Krakow, Poland; 4 MTM Solutions sp. z o.o., al. 29 Listopada 130, 31-406 Kraków, Poland; p.buda@mtmsolutions.pl

**Keywords:** historic wooden floorings, heritage conservation, floor renovation, risk management, environmental control

## Abstract

**Highlights:**

**What are the main findings?**

- A systemotechnical framework was applied to heritage wooden floor restoration.
- Risk levels evolved across restoration stages and were reduced through targeted mitigation.
- Environmental control and early diagnostics proved critical to intervention reliability.

**What are the implications of the main findings?**

- Risk assessment can function as a dynamic decision-support tool in heritage interiors.
- Modern materials can be integrated into historic floors under risk-aware planning.
- The approach is transferable to other heritage flooring interventions.

**Abstract:**

Decorative wooden floorings in heritage interiors require restoration strategies that balance material authenticity, technical reliability, and environmental sensitivity. This study presents a conservation-oriented restoration of a historic parquet floor in the Monastery at Kalwaria Zebrzydowska (Lesser Poland Voivodeship, Poland), originating from the late nineteenth and early twentieth centuries, and focuses on the role of structured risk analysis in technological decision-making. A systemotechnical framework was applied to analyse the restoration as a sequence of interrelated stages governed by material, structural, environmental, technological, and organisational subsystems. Qualitative and semi-quantitative risk classification was integrated with diagnostic investigation, workshop renovation, subfloor reconstruction, reinstallation, and post-intervention monitoring. The results show that dominant risk categories shift across stages and can be progressively reduced through targeted mitigation measures, particularly those addressing moisture variability, material compatibility, and organisational coordination. Early-stage diagnostics combined with active microclimate control proved critical to process reliability and long-term performance, enabling the retention of approximately 85% of the original wooden material. The findings demonstrate the broader applicability of phase-based, risk-informed decision-making in heritage conservation, offering a transferable framework for sustainable restoration of historic wooden floors across diverse cultural and climatic contexts.

## 1. Introduction

Decorative wooden floors, particularly those found in historic residences and public heritage buildings, constitute specialised components of architectural heritage buildings rather than independent heritage objects. Nevertheless, due to their material complexity, craftsmanship, and functional exposure, they can be analytically examined as distinct conservation subjects within the broader context of historic structures. Their significance extends beyond aesthetic or artisanal value: each floor embodies a historical record of materials, construction techniques, and regional craftsmanship practices. Understanding and preserving these layered testimonies of human creativity requires not only manual skill but also a scientific approach to analysis, diagnosis, and conservation. Over time, such wooden flooring systems are subject to progressive degradation arising from mechanical wear, moisture fluctuations, biological activity, and environmental agents. The resulting typical pathologies include surface abrasion and loss of decorative layers, cracking and splitting due to cyclic moisture changes, biological degradation caused by fungi or insects, detachment or delamination of parquet elements, permanent deformation such as cupping or warping, and structural instability resulting from subfloor settlement or joist deterioration [1,2,3]. Identifying these pathologies and their underlying causes is therefore fundamental not only for diagnostic purposes but also for defining risk factors that govern technological decision-making during conservation-oriented restoration.

Monitoring these processes of deterioration, and identifying the physical and chemical factors that accelerate them, are therefore crucial to defining effective and minimally invasive conservation strategies. Unlike standard flooring refurbishment, the restoration of historic wooden floors should balance material authenticity with technical feasibility. It demands an integrated methodology that combines analytical investigations, non-destructive diagnostic testing, and environmental assessment with traditional restoration techniques. Within this context, the study of degradation mechanisms and risk factors becomes fundamental to decision-making, ensuring that interventive actions are both scientifically informed and ethically sound—compare, e.g., [1,3].

Several research studies have addressed various aspects of timber flooring diagnostics and repair. Studies have proposed methods for structural inspection and defect classification [1,2], evaluated hybrid reinforcement solutions [4], and investigated the performance of protective coatings and adhesives aimed at improving durability [5,6]. In the field of heritage conservation, particular attention has been given to moisture control, material compatibility, and the influence of environmental conditions on the long-term stability of parquet panels [7,8]. Yet, despite these contributions, comprehensive approaches linking analytical assessment, technological restoration, and risk management remain scarce.

This paper addresses this gap by examining a conservation-oriented restoration of a decorative wooden floor in a heritage interior, analysed as a technically and organisationally complex process subject to multiple interacting risks.

The study integrates diagnostic investigation, process documentation, and phase-based risk classification within a systemotechnical framework. The main aim is to demonstrate how technological decision-making in heritage flooring restoration can be supported by structured, dynamic risk assessment rather than isolated qualitative judgement. The results show that dominant risks shift across successive stages of intervention and can be progressively reduced through targeted mitigation measures, particularly those related to moisture variability, material compatibility, and organisational coordination. By linking a clearly defined sequence of works with systemotechnical risk analysis, the study contributes a transferable methodological approach that supports reliable, conservation-led restoration of historic wooden interiors.

## 2. State-of-the-Art Review and Background

The restoration of decorative wooden floors in heritage buildings requires procedures that respond to the anisotropic, hygroscopic, and biologically sensitive nature of timber, while fulfilling the conservation imperative of preserving original materials and surface appearance. Within this domain, structured inspection-and-diagnosis frameworks remain fundamental, enabling defect typology, root-cause identification, and analytical mapping of degradation phenomena that guide interventions with minimal loss of historical fabric [1,2]. Beyond diagnosis, technological strategies include local or systemic replacement, scarfed and tongue-and-groove reconstructions, adhesive bonding schemes, and controlled surface re-machining, often supported by laboratory characterisation of material interfaces and coating performance [7,9]. The evolution of protective coating technologies, from traditional film-forming finishes to functional nanocomposite systems, has introduced new opportunities for improved mechanical and optical behaviour while enhancing resistance to wear and micro-abrasion [5]. Research on engineered wood constructions similarly informs dimensionally stable layups and bonding systems that can be adapted for conservation-oriented renovation [10].

**Figure 1:**
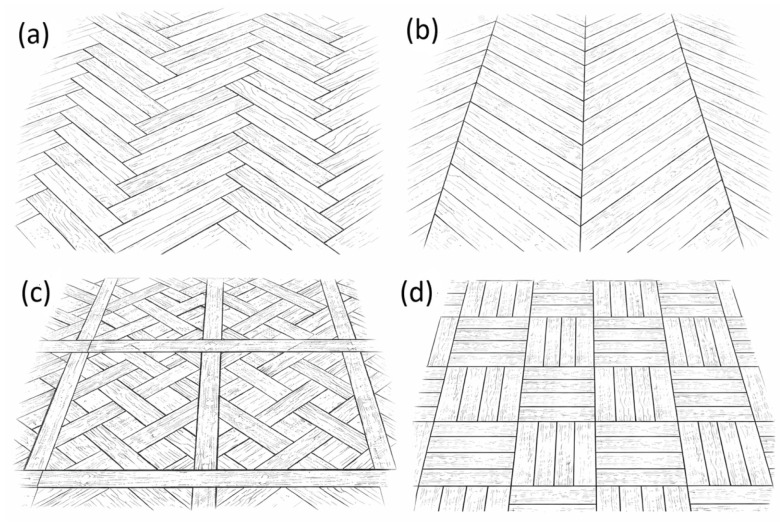
Sketches of common wooden flooring patterns: (**a**) herringbone; (**b**) chevron; (**c**) parquet de Versailles; (**d**) basket weave. (Source: own study).

**Figure 2:**
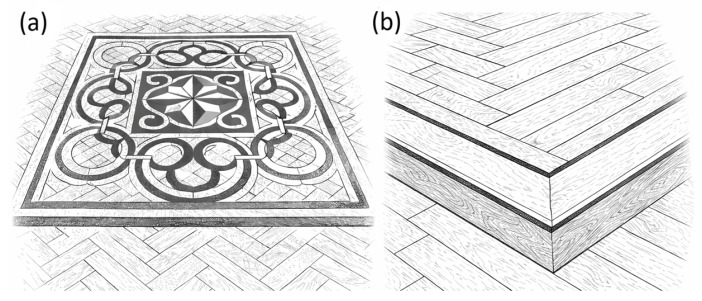
Sketches of wooden flooring exemplary decorations: (**a**) patterned inlay; (**b**) bordered flooring. (Source: own study).

Figure 1 illustrates representative patterns of historic decorative wooden floors, while Figure 2 presents examples of patterned inlay producing pictorial or geometric marquetry effects and contrasting borders, which traditionally served to frame and visually define the flooring field.

Material selection and application methods critically affect long-term performance and the durability of conservation outcomes. Service-life-oriented studies highlight the importance of specification accuracy and process control to mitigate premature ageing and degradation [11,12]. Conservation practice complements these approaches with targeted moisture regulation, microvoid and defect repairs, and biological hazard mitigation, including the use of selective antimicrobial or consolidation treatments, such as silver-ion-based systems applied locally to wood-based flooring components [13]. Where structural reinforcement is required, hybridisation techniques and sympathetic stiffening solutions have been explored—from discreet timber reinforcements and connection upgrades to wood–concrete composite systems—provided that reversibility, compatibility, and minimal intervention principles are observed [4,14]. More recent concepts have even investigated structural glass overlays in timber floors, offering stiffness enhancement with reduced mass and potential environmental benefits [15].

Before any interventive action, robust analytical assessment remains essential. Combined field and laboratory investigations clarify the in situ behaviour of floor assemblies and help discriminate between substrate-driven failures and defects originating from finishing or installation [3,16]. Numerical modelling, particularly finite-element approaches, further supports prediction of deflection, vibration, and the effects of lateral reinforcement or discontinuities [17], while more recent applied-science studies emphasise the value of structured analytical and decision-support frameworks for managing complex technical processes under uncertainty [18].

Parallel advances in environmental diagnostics have linked the degradation of historic timber floors to volatile organic compound (VOC) emissions, temperature-dependent off-gassing, and poor ventilation during conservation works [19,20,21]. Underfloor heating and fluctuating hygrothermal regimes introduce additional stresses, making thermal and moisture compatibility critical design parameters. Related studies underline the importance of selecting species, build-ups, and finishes that mitigate differential movement and cupping [8,22].

The literature thus converges on a multidomain risk framework governing heritage floor renovation, in which material, environmental, technological, and organisational factors are treated as interdependent components of a complex conservation system—compare with [23]. Technical risks encompass moisture mismeasurement, adhesive incompatibility, overaggressive surface treatment, and mismatches between historic timbers and modern materials [1,3]. Material risks involve biological deterioration, scarcity of compatible timber species, colour-matching difficulties, and the loss of material authenticity [9,11]. Organisational risks stem from incomplete documentation, sequencing conflicts, or insufficient coordination with conservation authorities [14]. Environmental risks (notably fluctuations in relative humidity, temperature, and ultraviolet exposure) amplify post-rehabilitation movement or finish failure [8,15]. Finally, health and safety risks arise from airborne pollutants and emissions during treatment; current best practice therefore promotes low-emission systems and active VOC monitoring throughout renovation [19,20,21]. Comparative analyses between restoration and new-build conditions confirm that heritage renovation projects are inherently more exposed to uncertainty, labour-intensive fitting, and substrate-related contingencies—insights consistent with prior syntheses of palace-type parquet floors and their executional challenges.

Beyond the engineering domain, decision-making in conservation reflects broader social and environmental dimensions. Architects and heritage professionals increasingly consider material provenance, certification, and sustainability indicators as determinants of acceptability and value [24,25]. Life-cycle assessments comparing flooring materials reinforce the environmental and cultural merit of conserving existing floors where feasible [26]. Although fire-performance studies remain peripheral to decorative conservation, they define important boundary conditions for adaptive re-use in public or mixed-use contexts [27,28]. Heritage-specific research, such as that conducted on the Kalwaria Zebrzydowska complex, demonstrates how technological choices should be integrated with cultural-landscape value assessments and statutory conservation regimes [29,30].

Against this background, the present article consolidates a technology-centred yet analytically grounded account of decorative wooden floor restoration and couples it with a structured, risk-based framework tailored to heritage interiors. It integrates diagnostic observation, process documentation, and analytical reasoning to derive process guidance that contributes to both the scientific study of cultural heritage objects and the advancement of conservation procedures. Particularly in Poland, heritage conservation is governed and guided by law [31], which establishes a multi-level system of legal protection. The protection levels entail differentiated legal requirements and approval procedures that influence the scope and methods of works.

## 3. Methodological Framework

The analysis and management of risks in the restoration of historic wooden floors require a structured, adaptive, and scientifically informed framework. Unlike new construction projects, conservation interventions are characterised by significant uncertainty, resulting from the age and heterogeneity of materials, undocumented past alterations, and the strict requirement to preserve historical authenticity. In such contexts, purely quantitative methods are often infeasible due to incomplete or non-comparable data; consequently, qualitative and semi-quantitative analytical approaches provide a more suitable basis for risk identification and evaluation.

To address these challenges, the present study adopts the systemotechnical approach formulated by Kutut and Ustinovicius [32], originally developed for the integrated assessment of cultural heritage management processes. This method allows the restoration of decorative wooden floors to be analysed as a multilevel system in which technological, material, environmental, and organisational factors interact dynamically. The approach enables the decomposition of a complex conservation process into interrelated subsystems and supports decision-making through structured evaluation of criteria at different hierarchical levels.

Within this framework, the systemotechnical approach is understood as a general analytical logic for the assessment and management of complex cultural heritage processes, rather than as a prescriptive, object-specific procedure. It is based on the interpretation of cultural heritage as a system composed of interacting physical, technological, environmental, organisational, and managerial components, whose relative significance and internal relationships depend on the scale and character of the object under consideration. As a result, the approach may be applied to individual elements of historic buildings, such as decorative wooden floors, as well as to broader subsystems or entire heritage structures, provided that the system boundaries, evaluation criteria, and risk indicators are appropriately adapted to the specific case. In this study, the systemotechnical methodology is therefore operationalised at the level of a single wooden flooring as a representative component of a historic building, demonstrating its applicability following case-specific adjustment rather than universal, unmodified transfer.

### 3.1. Systemotechnical Logic

According to the systemotechnical methodology, the object of analysis, in this case, a heritage flooring restoration process, is treated as a system of interdependent subsystems, each characterised by specific functions and risk parameters. The system operates within a specific environment and is influenced by feedback mechanisms between its elements.

The overall procedure consists of several key stages:

- Diagnostic modelling—defining system boundaries, identifying the physical, technological, and environmental characteristics of the wooden floor, and collecting diagnostic and contextual data;
- Hierarchical structuring—dividing the system into subsystems: material, structural, environmental, technological, and organisational;
- Qualitative evaluation—assessing each subsystem using verbal or ordinal scales (e.g., low–medium–high) based on expert knowledge, analytical findings, and environmental monitoring data;
- Integration and optimisation—synthesising results to identify dominant risks, critical interdependencies, and potential areas for process improvement;
- Feedback and refinement—incorporating post-intervention observations and monitoring data to update the model and improve predictive reliability.

This structured workflow supports the systematic interpretation of risks and ensures that analytical findings can be directly linked to decision-making processes.

The systemotechnical approach that was adapted from [32] is formulated as a structured, iterative methodology for the analysis and management of heritage wooden floor restoration. The procedure commences with diagnostic modelling, which establishes the initial technical state of the object and identifies the principal factors governing its performance. This diagnostic output provides the formal basis for hierarchical structuring and subsequent subsystem evaluation.

The methodology is organised around five interacting subsystems: material, structural, environmental, technological, and organisational. Each subsystem is evaluated using defined criteria, while interdependencies between subsystems are explicitly considered to capture combined effects influencing restoration decisions. This subsystem-based analysis constitutes the core analytical stage of the approach.

Results obtained from subsystem evaluations are synthesised through integration and optimisation procedures, enabling the formulation of technically and organisationally coherent intervention strategies. The methodology incorporates systematic feedback mechanisms whereby data from implementation, monitoring, and post-intervention assessment are reintroduced into earlier analytical stages.

This feedback process allows diagnostic assumptions to be verified and adjusted, subsystem parameters to be recalibrated, and decision criteria to be progressively refined on the basis of empirical evidence. Consequently, the methodology operates as a learning system, enhancing the reliability of predictions and the adequacy of proposed interventions across successive iterations.

Within this methodological framework, the material subsystem provides the most direct response to observed changes in wood condition and surface protection performance, while environmental and organisational subsystems supply contextual constraints affecting process stability and resource allocation. By formally embedding feedback-driven updates, the systemotechnical approach supports reproducible, evidence-based conservation aligned with contemporary standards in cultural heritage management.

### 3.2. Hierarchical Model for Risk Classification and Risk Matrix

Within the systemotechnical framework, risks are evaluated through a hierarchical model that distinguishes between general categories and specific sub-criteria. At the first level, four principal risk groups are proposed:

1. Technical and technological risks—associated with the reliability of diagnostic data, material compatibility, workmanship precision, and the adequacy of applied construction methods.
2. Organisational and managerial risks—linked to scheduling, communication between project stakeholders, coordination of specialist tasks, and documentation control.
3. Economic and financial risks—covering constraints of budget allocation, cost growth, procurement limitations, and availability of compatible materials.
4. External and environmental risks—encompassing climatic instability, ecological and legal constraints, and broader socio-political or regulatory conditions influencing project delivery.

Each group can be decomposed into detailed indicators, for example, availability of skilled conservators, accuracy of diagnostic documentation, humidity control precision, or reversibility of applied materials. Evaluation is typically expressed through verbal or ordinal scales, enabling comparison where quantitative data are lacking and providing a transparent basis for expert judgement.

Building upon the systemotechnical model, a five-step algorithm is applied for risk-oriented decision-making in heritage flooring projects:

1. Definition of objectives and stakeholder expectations—clarifying the purpose, scope, and desired conservation outcomes.
2. Collection of contextual information—gathering diagnostic, historical, and environmental data relevant to system definition.
3. Identification of internal and external risk indicators—defining technical, material, and organisational parameters influencing system behaviour.
4. Hierarchical evaluation of probability and impact—assigning qualitative ratings through expert consensus or analytical observation.
5. Selection and prioritisation of mitigation strategies—choosing risk mitigation measures based on criticality, feasibility, and reversibility.

To facilitate application of the approach for the restoration of wooden heritage floorings, a generalised risk matrix is proposed in Table 1. The matrix captures the principal relevant risk domains, rated by likelihood and impact according to a standard qualitative scale.

This classification enables a systemic and comparative assessment of restoration risks, supporting the prioritisation of interventions that balance material authenticity, safety, and long-term stability. The systemotechnical framework thus integrates analytical investigation, conservation management, and risk-based reasoning into a single methodological platform applicable to diverse heritage flooring contexts.

## 4. Restoration of the Historic Wooden Flooring in the Monastery at Kalwaria Zebrzydowska

The Sanctuary and Monastery at Kalwaria Zebrzydowska (located in Lesser Poland Voivodship, Poland), founded in the early seventeenth century by Mikołaj Zebrzydowski, form part of a Mannerist architectural and landscape complex inscribed on the UNESCO World Heritage List—see, e.g., [30].

**Figure 3:**
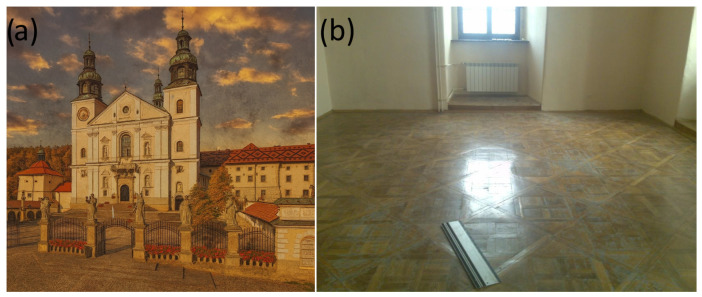
(**a**) View of the Monastery in Kalwaria Zebrzydowska. (**b**) Monastery room interior and general view of the flooring before restoration. (Author: Piotr Buda).

A general view of the Monastery is presented in Figure 3a. Within this ensemble, one of the rooms in the Monastery contained Versailles-type parquet flooring that had suffered severe degradation. A view of this room is presented in Figure 3b.

**Figure 4:**
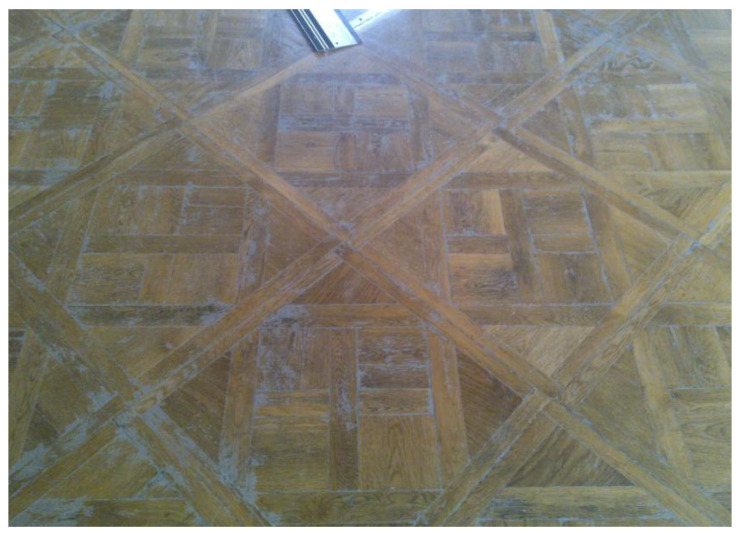
Detailed view of the flooring before restoration. (Author: Piotr Buda).

A detailed view of the flooring before the restoration is presented in Figure 4. The flooring exhibited local delamination, loss of surface finish, and deformation. The probable causes of this condition were ageing, uneven settlement of the timber joists, structural movements, and previous repairs using incompatible materials.

Because of the historical and heritage value of the floor, a conservation-oriented restoration approach was adopted. The project therefore provided an opportunity to analyse the technological sequence and the risks associated with heritage conservation interventions.

### 4.1. Restoration Process in Accordance with Systemotechnical Logic and Approach

The restoration of the historic wooden flooring was carried out as a clearly defined sequence of technological operations, structured to progressively reduce uncertainty and control risks inherent to heritage interventions. Each stage was planned and executed in accordance with the systemotechnical framework described in Section 3, ensuring that diagnostic findings, technological decisions, environmental constraints, and organisational requirements were consistently integrated throughout the process.

The first stage comprised diagnostic investigation and planning. Detailed inspection of the floor and its supporting structure was carried out through local openings, allowing identification of the subfloor build-up and assessment of timber joists laid on compacted sand beneath a blind boarding layer. Instrumental moisture measurements using the CM-Garet method revealed pronounced spatial variability, indicating insufficient ventilation of the substrate. Concurrently, visual condition mapping documented cracking, surface abrasion, and biological degradation of selected elements. Investigation revealed that the original flooring was manufactured from oak wood. The parquet panels were produced at the turn of the nineteenth and twentieth centuries in a craft workshop located in the territory of present-day Germany. Each individual panel was labelled and numbered by the manufacturer. All parquet panels were photographically recorded and systematically numbered prior to dismantling. Completion of this stage established the baseline condition of the system and defined the initial risk profile governing subsequent operations. For clarity, the basic properties of oak wood that are relevant to the performance and conservation of historic wooden flooring are summarised in Table 2.

Based on the diagnostic investigation and planning stage, a set of planned materials and technological operations was defined. The renovation strategy assumed maximum retention of original oak elements, with local replacement limited to components affected by irreversible biological degradation. Replacement and supplementary elements were planned to be manufactured from oak and smoked oak. Planned operations included workshop-based repair and reformatting of original panels, reconstruction of missing decorative elements, and on-site reinstallation on a newly prepared subfloor. Bonding was planned using synthetic resin-based adhesives, while minor cracks and surface defects were to be repaired using natural resin-based fillers. Surface finishing was planned to involve sequential sanding with progressively finer abrasives (36, 60, and 100 grit), followed by final smoothing and protective finishing using a staining system, primer lacquer, and a two-component topcoat to ensure durability and abrasion resistance.

**Figure 5:**
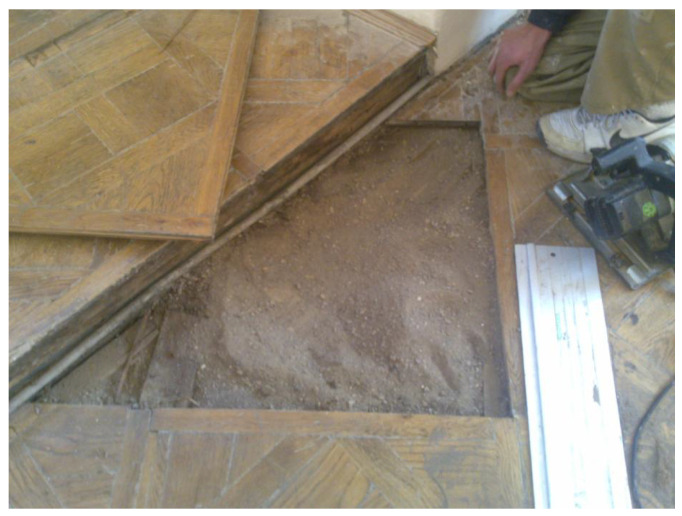
Dismantling of the flooring panels. (author: Piotr Buda).

The second stage involved controlled dismantling and inventory of the parquet panels. Dismantling was performed in a manner that preserved the integrity of individual panels and exposed concealed structural components for verification. The numbering system ensured traceability and enabled exact reconstruction of the historical layout. This stage functioned as a transition between diagnostic modelling and technological intervention, reducing uncertainty related to hidden defects and confirming the scope of necessary repairs. Figure 5 presents the dismantling of the flooring panels. Based on the systematic dismantling, numbering, and inventory of all parquet panels, it was established that approximately 85% of the original wooden material was suitable for conservation and reuse. This assessment was carried out by the authors through direct inspection and verification of individual elements, including evaluation of structural integrity, dimensional stability, and surface condition. The remaining elements required partial reconstruction due to irreversible degradation or loss.

**Figure 6:**
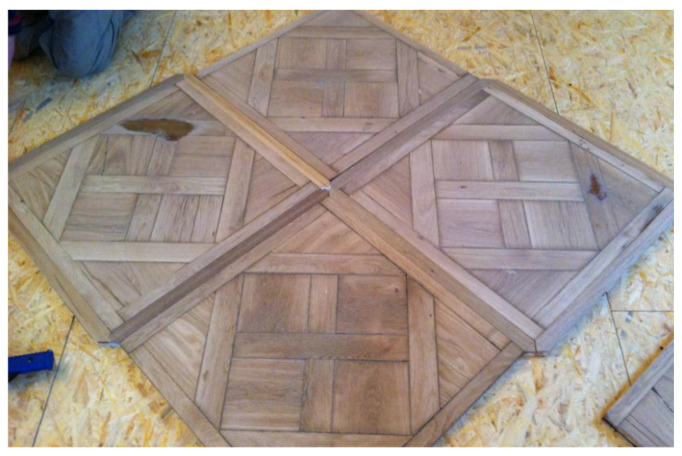
Trial preinstallation of renovated panels. (author: Piotr Buda).

The third stage consisted of workshop restoration of parquet panels, representing the primary technological phase of the sequence. Each panel underwent dimensional verification, revealing long-term deformation requiring geometric correction. Panels were reformatted to uniform dimensions while retaining original composition, followed by controlled sanding to equalise usable thickness. Tongue-and-groove joints were cleaned manually, missing decorative elements were reconstructed using oak wood selected for material and mechanical compatibility with the original, naturally aged flooring, and minor cracks were repaired with natural resin-based fillers. Areas of material loss were reconstructed using smoked oak elements selected for their material compatibility and visual coherence with the original flooring. This stage concluded only after verification that geometric, material, and visual criteria were satisfied, ensuring readiness for reinstallation. Figure 6 illustrates the trial preinstallation of renovated panels for geometric verification.

The fourth stage addressed reconstruction of the subfloor, undertaken prior to any on-site reinstallation. Deteriorated joists and sand bedding were removed and replaced with a new ventilated load-bearing grid. A sealed blind floor was installed to stabilise the substrate and regulate moisture transfer. Completion of this stage provided a structurally and environmentally controlled base, a prerequisite for durable reinstallation of the restored panels.

**Figure 7:**
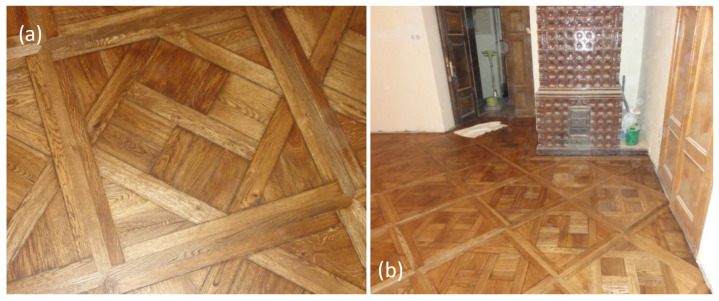
Restoration results. (**a**) Detailed view of the panel. (**b**) Monastery room interior and general view of the flooring after restoration (author: Piotr Buda).

The fifth stage comprised on-site reinstallation and surface finishing. Restored panels were installed according to the historical diagonal layout and bonded using an elastic adhesive compatible with fluctuating indoor humidity. Manual fitting ensured continuity of joints and decorative alignment across the entire floor. After a stabilisation period, surface finishing was carried out through multi-stage sanding, staining, and application of a low-emission, water-borne protective system. Environmental conditions were actively controlled throughout this stage to ensure proper curing of adhesives and finishes. Figure 7 presents results of the restoration process.

The final stage involved post-intervention verification and monitoring, closing the systemotechnical loop. Mechanical performance testing and visual assessment confirmed stable adhesion, uniform stiffness, and compliance with functional and aesthetic requirements. The majority of the original material was retained, and all process data were archived to support future maintenance and potential HBIM (Heritage Building Information Modelling) integration. Feedback from this stage was formally incorporated into the analytical framework, enhancing the predictive reliability of future conservation interventions.

To clarify the relationship between the sequence of works and the applied systemotechnical logic, the individual restoration stages were analysed in terms of the dominant and interacting subsystems governing system behaviour at each phase. This mapping highlights how subsystem influence shifts over time, reflecting changes in technical objectives, risk exposure, and decision-making priorities.

Table 3 presents a structured correspondence between the restoration stages and the material, structural, environmental, technological, and organisational subsystems, providing a concise overview of subsystem dominance and interaction across the entire intervention process.

### 4.2. Risk Classification and Analysis

Risk classification and analysis were conducted as an integral component of the restoration process, in accordance with the hierarchical model and qualitative risk matrix defined in Section 3.2 (Table 1). Rather than being applied as a one-time assessment, the risk matrix functioned as a dynamic analytical tool, updated sequentially as the project progressed through successive stages. This phase-based evaluation corresponds directly to the shifting subsystem dominance identified in Table 3.

During the initial diagnostic phase, technical and environmental risks were assessed as high. The principal risk drivers included pronounced spatial variability in moisture content, insufficient subfloor ventilation, uncertainty regarding concealed structural elements, and incomplete historical documentation. These factors increased both the likelihood and potential impact of inappropriate technological decisions. In response, mitigation strategies focused on extending instrumental diagnostics, stabilising the microclimate, and refining system assumptions through specialist consultation. As a result, risk levels associated with diagnostic uncertainty were reduced from high to medium prior to dismantling.

In the dismantling phase, organisational and material risks became dominant. The primary concerns related to loss of traceability, damage to original elements, and errors in sequencing that could compromise accurate reassembly. These risks were initially assessed as medium to high but were effectively mitigated through systematic numbering, photographic documentation, and conservation supervision. The application of controlled dismantling techniques reduced the likelihood of irreversible material loss, lowering residual risk levels to medium.

During workshop restoration, technological and material risks reached their highest relative significance. Key risk factors included excessive removal of original material during machining, adhesion incompatibility arising from heterogeneous fibre orientation, and visual discrepancies between original and reconstructed elements. These risks were initially classified as medium to high. The systemotechnical framework enabled iterative risk control through test samples, gradual processing, and continuous verification against predefined geometric and aesthetic criteria. By the completion of this phase, the residual risk level was reduced to low to medium, reflecting stabilised technological parameters.

The reconstruction of the subfloor and subsequent on-site reinstallation represented the phase with the highest combined environmental and organisational risk exposure. Fluctuations in indoor relative humidity directly affected curing processes, while restricted access to the heritage interior increased coordination complexity. These risks were assessed as high at the outset of this stage. Mitigation measures included temporary climate control, adaptive scheduling, and continuous on-site supervision. The effectiveness of these measures was confirmed by the absence of adhesion failures or geometric instability, reducing final risk levels to medium.

In the final phase, residual risks across all categories were reassessed based on post-intervention monitoring and performance verification. Mechanical testing and visual inspection confirmed stable adhesion, uniform stiffness, and compliance with functional and aesthetic requirements. At this stage, all major risk categories were classified as low, with remaining uncertainties limited to long-term environmental behaviour typical of historic interiors. The systematic archiving of diagnostic and process data further reduced organisational and informational risks related to future maintenance.

Across the full renovation sequence, the application of the risk matrix demonstrated clear risk migration between categories and a progressive reduction in overall risk levels. The most critical risk drivers were associated with moisture variability and organisational coordination, confirming the strong influence of the environmental and organisational subsystems identified in Table 3. The case study confirms that embedding qualitative risk assessment within a systemotechnical framework enables informed, adaptive decision-making, supporting both material authenticity and long-term functional reliability.

To synthesise the phase-based risk assessment described above, the evolution of dominant risk categories and the effectiveness of applied mitigation measures are summarised in Table 4.

The table consolidates the qualitative risk ratings assigned at the beginning and end of each restoration stage, highlighting the dynamic nature of risk within the systemotechnical framework. By presenting risk reduction as a function of sequential interventions, Table 4 illustrates how targeted mitigation strategies progressively lowered overall risk levels while supporting informed decision-making throughout the restoration process.

**Figure 8:**
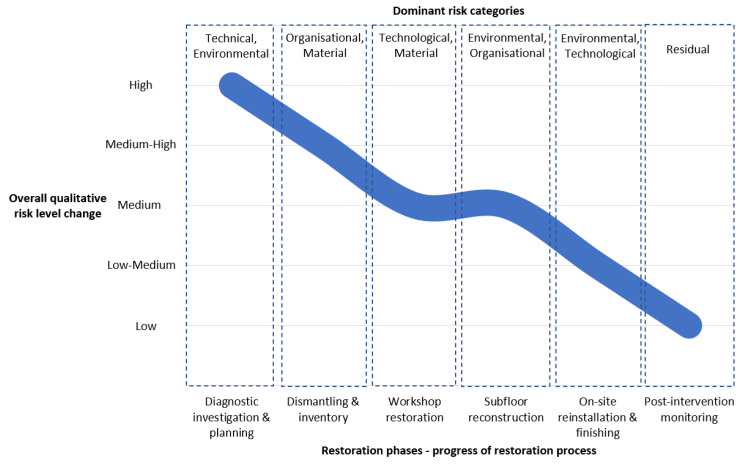
Infographic illustrating the qualitative trajectory of overall project risk across the restoration process of the studied case of wooden flooring. (Source: own study).

To synthesise the results of the phase-based risk evaluation, Figure 8 presents a graphical interpretation of the evolution of overall project risk across successive stages of the restoration process. The curve illustrates progressive risk reduction as mitigation measures are implemented, while the labels above indicate the dominant risk categories influencing decision-making at each phase within the systemotechnical framework. This representation highlights both the non-linear character of risk evolution and the migration of dominant risk drivers as the intervention progresses, from early-stage technical and environmental uncertainties through organisational and technological challenges during execution to residual risks associated with long-term performance. By complementing the detailed tabular analysis, the figure provides an integrated overview of how structured diagnostics, controlled technological interventions, and environmental management collectively contributed to the stabilisation of the restoration process.

## 5. Discussion

The renovation of decorative wooden floors in heritage interiors represents a complex, multi-dimensional process in which conservation ethics, construction technology, environmental control, and organisational coordination intersect. The case of the palace parquet floor at Kalwaria Zebrzydowska demonstrates that technical success cannot be attributed solely to craftsmanship or material selection; rather, it depends on the ability to manage interdependencies between system components across a clearly defined sequence of works. This observation is consistent with system-oriented perspectives on heritage conservation, which emphasise the need to integrate material, environmental, technological, and organisational dimensions within a unified analytical framework [23]. The results of this study therefore provide both confirmation of existing research and methodological advancement in the risk-oriented management of heritage flooring restoration. From a technological perspective, the sequential structure of the renovation process closely corresponds to the multi-phase approaches proposed in [1,2], which emphasise systematic inspection, defect classification, and staged intervention. The diagnostic investigation conducted at Kalwaria Zebrzydowska confirms that early identification of moisture variability, subfloor irregularities, and concealed degradation significantly reduces the likelihood of secondary failures at later stages. Consistent with the findings in [3,16], the integration of instrumental measurements with visual mapping provided a reliable basis for planning. However, the present study extends these approaches by embedding diagnostic outputs within a dynamic risk framework, allowing diagnostic assumptions to be continuously updated as the project progresses.

The workshop restoration phase highlights the technological duality frequently discussed in the conservation literature, in which traditional craftsmanship should be reconciled with modern materials and processing methods. As noted in [14], such hybridisation introduces risks related to material compatibility and authenticity. In the Kalwaria case, controlled re-formatting of panels, conservative sanding strategies, and the use of compatible smoked oak inserts enabled geometric correction while preserving visual coherence. The successful application of low-emission resin-based adhesives supports the “balanced innovation” principle described in [4], providing empirical evidence that contemporary materials can be safely integrated into historic interiors when their use is governed by iterative testing and risk control. The effectiveness of conservation and renovation interventions in wooden heritage structures depends strongly on the practitioner’s material knowledge and experience, particularly with respect to the mechanical behaviour, durability, and functional suitability of different wood species, as demonstrated in studies of historic wooden mechanisms and structures [33].

In this context, the role of the conservator’s professional expertise extends beyond manual skill to include the ability to interpret diagnostic data, anticipate material behaviour under variable environmental conditions, and select intervention techniques that balance structural performance with conservation ethics. In the Kalwaria Zebrzydowska case, informed judgement was essential in determining acceptable tolerances for material removal, selecting compatible replacement elements, and sequencing operations to minimise irreversible impacts. These decisions illustrate how practitioner expertise functions as a critical risk-mitigating factor within the restoration process, directly influencing both technical durability and heritage authenticity.

Environmental conditions emerged as a dominant risk driver across several stages of the restoration sequence. In line with observations presented in [8,22], fluctuations in relative humidity directly affected adhesive curing and dimensional stability. The case study confirms that environmental control cannot be treated as a passive background condition; instead, it functions as an active technological parameter influencing both process timing and material performance. The implementation of temporary climate stabilisation measures and continuous monitoring aligns with the integrated structural–environmental perspective advocated in [15], reinforcing the necessity of treating environmental management as an integral component of conservation technology.

A key contribution of the present study lies in the application of the systemotechnical risk framework as a decision-support tool rather than a purely descriptive assessment. Unlike conventional qualitative evaluations, the phase-based risk classification enabled systematic prioritisation of threats and mitigation measures at each stage of the restoration process. As demonstrated in Table 4, risk levels migrated between categories and were progressively reduced through targeted interventions. This structured approach facilitated communication between engineers, conservators, and contractors, confirming the relevance of integrated management concepts discussed in [34] at the scale of interior heritage interventions.

Organisational factors proved equally critical to project outcomes. The systematic documentation of dismantling, restoration, and reinstallation phases, combined with the creation of a digital photographic archive, enhanced traceability and transparency. The proposed integration of these records into an HBIM environment aligns with current trends in heritage digitalisation [29,30] and supports the transformation of renovation from a single, isolated intervention into a long-term management process informed by documented risk history.

Finally, the results contribute to ongoing discussions on sustainability in architectural conservation. The retention of approximately 85% of the original oak material preserved historical authenticity while reducing material consumption and embodied environmental impact. This outcome supports lifecycle-oriented perspectives on flooring restoration advanced by the studies [12,26], demonstrating that conservation-led approaches can simultaneously fulfil cultural, technical, and environmental objectives when guided by structured risk-aware planning.

In summary, the Kalwaria Zebrzydowska case confirms that risk management provides a unifying framework for coordinating technological, environmental, organisational, and conservation dimensions in heritage wooden flooring restoration. By combining a clearly defined sequence of works with systemotechnical risk analysis and expert-led technological decision-making, the study demonstrates how modern diagnostic methods, adaptive material use, and environmental control can be integrated within traditional conservation principles to achieve outcomes that are both technically durable and culturally authentic. The principal contribution of this study lies in the operationalisation of a systemotechnical risk framework within a clearly sequenced heritage flooring restoration process. While previous research has addressed individual aspects of diagnostics, material compatibility, or environmental control, this work demonstrates how risk assessment can function as a dynamic decision-support mechanism across successive technological stages. By explicitly linking subsystem dominance, risk migration, and mitigation outcomes, the proposed approach advances existing conservation methodologies from static, phase-isolated evaluations toward an integrated, feedback-driven model. The case study confirms that qualitative and semi-quantitative risk tools, when embedded within a systemotechnical logic, can effectively guide technological choices, coordinate interdisciplinary actors, and reduce uncertainty in small-scale heritage interventions, thereby extending the applicability of risk-based management beyond large infrastructural or landscape-scale conservation projects.

## 6. Conclusions

The restoration of decorative wooden floors in heritage interiors is characterised by high technical complexity, environmental sensitivity, and organisational uncertainty. The case of the palace parquet floor in the Monastery at Kalwaria Zebrzydowska demonstrates that successful conservation outcomes depend on the structured coordination of diagnostic investigation, technological intervention, environmental control, and risk management across a clearly defined sequence of works. The study confirms that comprehensive early-stage diagnostics are essential for identifying concealed structural irregularities and moisture variability that cannot be detected through surface inspection alone. Integrating diagnostic findings into subsequent decision-making significantly reduced the risk of secondary failures during later restoration stages.

The results further show that balanced technological adaptation enables the safe integration of contemporary materials and techniques into historic flooring systems. Elastic resin-based adhesives, low-emission coating systems, and engineered moisture-control layers proved compatible with original materials when applied conservatively and verified through controlled testing. This confirms that modern technologies can support conservation objectives when governed by risk-aware planning.

Environmental conditions emerged as a decisive factor influencing process reliability and long-term performance. Active control and monitoring of indoor microclimate, particularly relative humidity, were critical to ensuring proper curing behaviour and dimensional stability, demonstrating that environmental management should be treated as an integral technological parameter rather than a secondary condition.

A central contribution of this work lies in the operational application of systemotechnical risk management as a dynamic decision-support tool. The phase-based risk classification enabled systematic prioritisation and mitigation of dominant risks as they migrated between categories throughout the restoration process, improving coordination between stakeholders and transparency of decision-making.

Finally, the retention of approximately 85% of the original oak material confirms that conservation-led restoration can simultaneously preserve cultural authenticity and reduce environmental impact. The findings demonstrate that risk-based, systemotechnical planning provides a transferable and effective framework for managing uncertainty in heritage wooden flooring restoration.

Future research should focus on complementing the qualitative framework with quantitative performance indicators and validating the approach through comparative studies across different heritage flooring typologies.

Although the present study focuses on historic wooden flooring, the systemotechnical framework employed is not inherently limited to flooring systems. The methodology addresses the restoration process as a structured system composed of interacting material, structural, environmental, technological, and organisational subsystems. As such, it may be applied to other wooden components of historic buildings, including ceilings, staircases, wall panelling, or roof structures, provided that the system boundaries, evaluation criteria, and risk indicators are adapted to the specific material configuration, functional role, and conservation constraints of the object under consideration. The proposed approach should therefore be understood as a transferable decision-support framework rather than a universally prescriptive model.

## Figures and Tables

**Table 1:** Universal risk classification matrix for heritage wooden flooring restoration. (Source: own study).

Risk Category	Representative Factors	Example Indicators	Likelihood	Impact
Technical /Technological	inadequate diagnostics; incompatible adhesives; excessive sanding; tool calibration errors	accuracy of inspection; adhesion reliability	Low–High	Moderate–Severe
Material	Biological decay; concealed defects; limited availability of compatible timber; moisture mismatch	Moisture equilibrium; biological resistance	Low–High	Moderate–Severe
Environmental	RH and temperature fluctuations; microclimatic instability; UV degradation	Indoor RH variation; microclimate control	Low–High	Moderate–High
Organisational /Managerial	Scheduling errors; documentation gaps; inadequate supervision; poor coordination	Quality control procedures; supervision rate	Low–Medium	Low–High
Economic /Financial	Material cost volatility; funding delays; procurement risks	Budget stability; supplier reliability	Low–Medium	Moderate–High
Health and Safety	VOC emissions; dust exposure; fire risk during restoration	Air quality monitoring; compliance with standards	Low–Medium	Moderate–High

**Table 2:** Basic properties of oak wood relevant to the historic flooring renovation. (Source: own study).

Property	Oak Wood Characteristics	Relevance to Flooring Renovation
Density	High (typically 650–750 kg/m^3^ at 12% moisture content)	Provides good load-bearing capacity, wear resistance, and long-term mechanical stability under intensive use
Hygroscopic behaviour	Moderate to high hygroscopicity; anisotropic swelling and shrinkage	Requires strict control of indoor relative humidity to minimise dimensional movement and deformation
Durability	High natural durability; good resistance to biological degradation	Suitable for long-term use in historic interiors with controlled environmental conditions
Compatibility with adhesives	Good compatibility with elastic resin-based and modern conservation-grade adhesives	Allows secure bonding while accommodating moisture-induced movement
Compatibility with finishes	Good adhesion of traditional oil-based and modern low-emission water-borne coatings	Enables preservation of historical appearance while meeting contemporary environmental standards
Suitability for historic interiors	Historically authentic and widely used in palace and ecclesiastical flooring	Supports material authenticity, reversibility principles, and visual continuity in conservation practice

**Table 3:** Mapping of restoration phases to systemotechnical subsystems.

Restoration Phase	Material Subsystem	Structural Subsystem	Environmental Subsystem	Technological Subsystem	Organisational Subsystem
Diagnostic investigation and planning	Primary: assessment of wood condition, degradation, moisture content	Primary: evaluation of joists, subfloor composition, deformation	Primary: identification of moisture variability, ventilation deficits	Secondary: selection of diagnostic methods	Secondary: documentation, coordination with specialists
Dismantling and inventory	Primary: preservation of original material, traceability	Secondary: exposure of load-bearing elements	Secondary: temporary microclimate control	Primary: controlled dismantling techniques	Primary: sequencing, numbering, conservation supervision
Workshop restoration of panels	Primary: material conservation, repairs, patina matching	Secondary: correction of geometric deformation	Secondary: controlled workshop conditions	Primary: machining, sanding, reconstruction technologies	Secondary: quality control, documentation
Subfloor reconstruction	Secondary: compatibility of new materials	Primary: new load-bearing grid, stiffness and stability	Primary: ventilation design, moisture barriers	Primary: installation systems, bonding solutions	Secondary: scheduling, coordination of trades
On-site reinstallation and finishing	Primary: surface integrity, finish performance	Secondary: alignment and joint continuity	Primary: humidity and temperature control	Primary: adhesives, coatings, finishing processes	Primary: access management, supervision
Post-intervention monitoring and verification	Primary: material performance verification	Secondary: confirmation of structural behaviour	Primary: long-term microclimate stability	Secondary: performance testing methods	Secondary: archiving, maintenance planning

**Table 4:** Phase-based evolution of dominant risks and mitigation outcomes in the restoration process. (Source: own study).

Restoration Phase	Dominant Risk Categories	Initial Risk Level	Key Mitigation Measures	Residual Risk Level
Diagnostic investigation and planning	Technical, Environmental	High	Extended instrumental diagnostics; moisture mapping; temporary microclimate stabilisation; specialist consultation	Medium
Dismantling and inventory	Organisational, Material	Medium–High	Systematic numbering; photographic documentation; controlled dismantling; conservation supervision	Medium
Workshop restoration of panels	Technological, Material	Medium–High	Test samples; gradual machining; conservative sanding; colour-matching trials; full documentation of replacements	Low–Medium
Subfloor reconstruction	Environmental, Organisational	High	Ventilated subfloor design; moisture barriers; adaptive scheduling; continuous supervision	Medium
On-site reinstallation and finishing	Environmental, Technological	Medium	Climate control during curing; elastic adhesive systems; staged finishing	Low
Post-intervention monitoring and verification	Residual (all categories)	Low–Medium	Performance testing; visual inspection; digital archiving; maintenance planning	Low

## Data Availability

The original contributions presented in this study are included in the article. Further inquiries can be directed to the corresponding authors.
